# Endothelin-1 in the failing Fontan: pathobiology, precision therapeutics, and future trial design

**DOI:** 10.3389/fped.2025.1718057

**Published:** 2026-02-05

**Authors:** Iman Maiza

**Affiliations:** Macon & Joan Brock Virginia Health Sciences at Old Dominion University Eastern Virginia Medical School, Norfolk, VA, United States

**Keywords:** endothelin receptor antagonist (ERA), endothelin-1 (ET-1), Fontan circulation, Fontan-associated liver disease, precision medicine

## Abstract

The Fontan circulation, devised as definitive palliation for single-ventricle congenital heart disease, imposes systemic venous hypertension, loss of pulmonary arterial pulsatility, and restricted preload reserve. These hemodynamic trade-offs progressively injure the pulmonary vasculature, liver, and lymphatic system, producing late morbidities including elevated pulmonary vascular resistance, Fontan-associated liver disease (FALD), protein-losing enteropathy, and arrhythmias. Endothelin-1 (ET-1), a potent vasoconstrictor and profibrotic mediator, plausibly unifies these complications. Mechanistic studies demonstrate ET-1 upregulation in failed Fontan lungs, activating PLC–Ca^2+^, RhoA/ROCK, and MAPK/ERK cascades to drive vasoconstriction and remodeling. In cirrhotic livers, ET-1 localizes to stellate cells, promoting contraction and fibrogenesis, mechanisms biologically relevant to congestive FALD. Clinical cohorts consistently show elevated ET-1 correlating with hospitalization, exercise intolerance, and arrhythmias. Trials of endothelin-receptor antagonists (bosentan, ambrisentan, macitentan) demonstrate reassuring safety and suggest benefit when outcomes emphasize ventilatory efficiency or hepatic endpoints rather than peak oxygen consumption, which is physiologically constrained in Fontan physiology. Given the mixed results of existing trials, a framework is outlined that stratifies Fontan patients into pulmonary-inefficiency, congestive-hepatic, lymphatic, and arrhythmia-dominant phenotypes, using co-primary endpoints such as VE/VCO_2_ slope, elastography, and biomarker panels. By linking ET-1 biology to pragmatic trial design, this approach emphasizes targeted strategies that may stabilize the circulation, extend transplant candidacy, and improve long-term outcomes.

## Introduction

The Fontan circulation was developed as a definitive palliation for patients with single-ventricle congenital heart disease. In this physiology, systemic venous blood is directed to the pulmonary arteries without a subpulmonary ventricle. Advances in surgical techniques and perioperative management have improved survival, but the intrinsic hemodynamic trade-offs remain substantial. Chronic systemic venous hypertension, diminished pulmonary arterial pulsatility, and limited preload reserve impose progressive stress on the pulmonary vasculature, the liver, and the lymphatic system. Over time, these forces produce a constellation of late complications that include increasing pulmonary vascular resistance (PVR), Fontan-associated liver disease (FALD), protein-losing enteropathy, plastic bronchitis, and arrhythmias (1–3).

Endothelin-1 (ET-1) is a potent endogenous vasoconstrictor with established roles in vascular remodeling and fibrogenesis. In lung specimens obtained from patients with failing Fontan physiology, ET-1 and its receptors are markedly overexpressed, and circulating levels of ET-1 are frequently elevated after Fontan completion (4–7). These findings suggest that ET-1 may provide a mechanistic link between pulmonary vascular dysfunction and congestive hepatic injury in this population. This review synthesizes current knowledge of ET-1 biology within the Fontan circulation and evaluates the available clinical evidence across the lifespan. A critical appraisal of trials involving endothelin-receptor antagonists (ERAs) is provided, together with an examination of the limitations of peak oxygen consumption (VO_2_) as a primary endpoint. Finally, a precision-based framework for future trial design is proposed, aligning more closely with the pathobiology of the Fontan circulation.

## Endothelin biology and the fontan circulation

### Receptors and cellular specificity

Endothelin-1 acts primarily through the G-protein–coupled receptors ET_A_ and ET_B._ Activation of ET_A_ on pulmonary arterial smooth-muscle cells produces vasoconstriction and stimulates proliferation and extracellular matrix deposition, while smooth-muscle ET_B_ can mediate similar effects (4, 8–10). In contrast, endothelial ET_B_ receptors enhance nitric oxide and prostacyclin release and facilitate clearance of circulating ET-1, thereby serving a protective role (8, 9). In the Fontan circulation, where pulsatility is diminished and venous congestion is chronic, endothelial dysfunction likely reduces ET_B_ mediated protection and allows constrictor signaling to dominate (1, 2, 11).

### Downstream signaling

**Figure 1:**
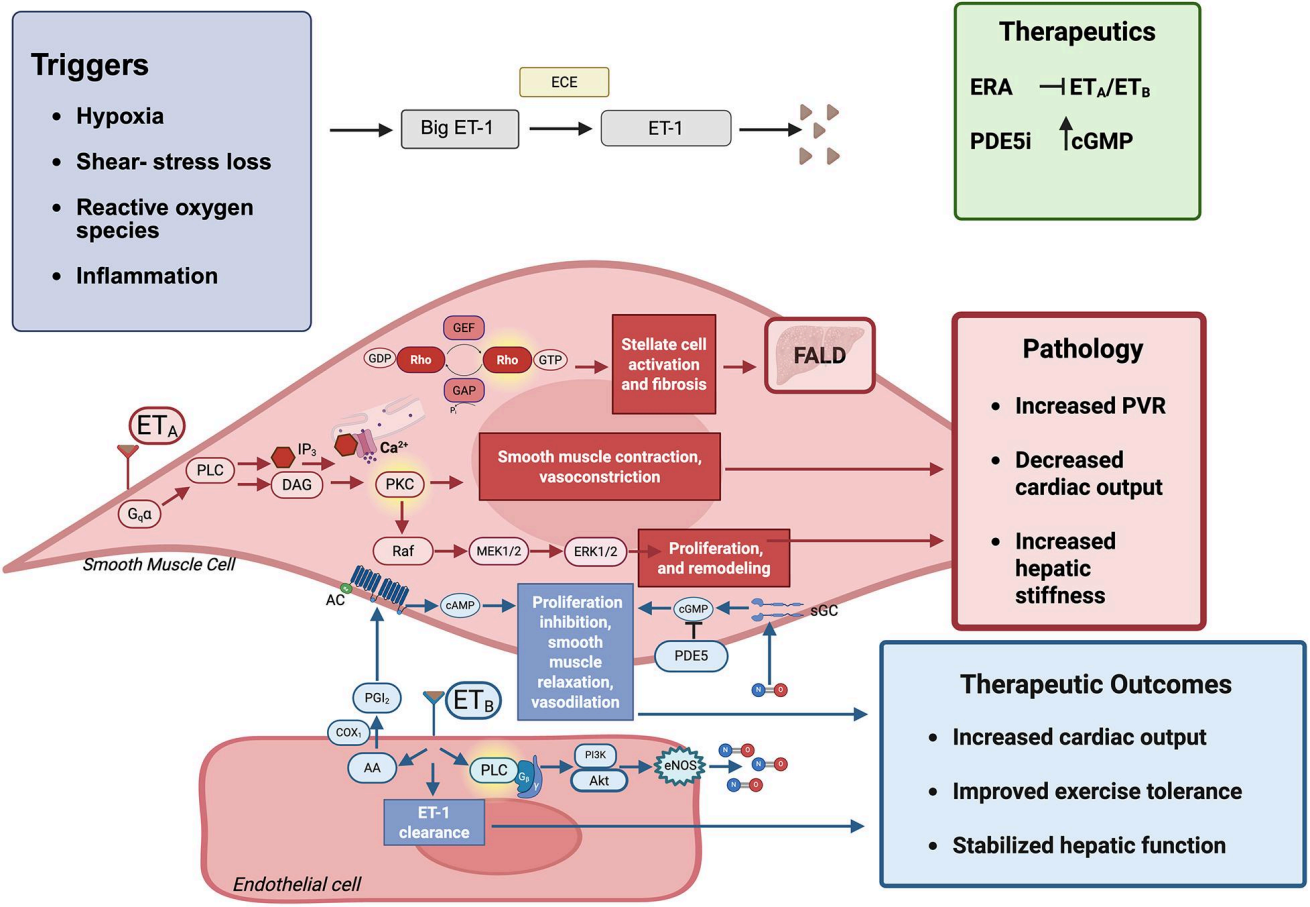
Schematic of endothelin-1 (ET-1) signaling in the Fontan circulation. Triggers such as hypoxia, shear-stress loss, reactive oxygen species, and inflammation increase ET-1 production. ET_A_ receptor activation in smooth muscle promotes vasoconstriction, proliferation, and fibrosis, contributing to elevated pulmonary vascular resistance (PVR), decreased cardiac output, and Fontan-associated liver disease (FALD, characterized by congestion, hepatic stiffness, and fibrosis). Endothelial ET_B_ signaling supports vasodilation and ET-1 clearance. Therapeutic strategies include endothelin receptor antagonists (ERAs, blocking ET_A_) and phosphodiesterase-5 inhibitors (PDE5i, preserving cGMP). Collectively, these approaches may increase cardiac output, stabilize hepatic function, and improve exercise tolerance.

ET_A_ receptor activation triggers phospholipase Cβ, leading to inositol trisphosphate and diacylglycerol generation and increased intracellular calcium, which raises vascular tone. Parallel activation of RhoA/Rho-kinase enhances calcium sensitivity, while MAPK/ERK pathways promote hypertrophy, proliferation, and matrix remodeling (4, 10, 12, 13). In experimental pulmonary hypertension, inhibition of Rho-kinase reverses ET-1–mediated vasoconstriction and attenuates remodeling, emphasizing the central role of this pathway (12, 13). Lung specimens from failed Fontan patients demonstrate increased expression of ET-1 and its receptors within intra-acinar arteries, accompanied by concentric medial hypertrophy and adventitial thickening, features that resemble pulmonary arterial hypertension (5). The central pathways through which ET-1 contributes to vascular tone and remodeling, as well as potential therapeutic targets, are summarized schematically in Figure 1.

### Regulation of ET-1 expression

Several stimuli relevant to the Fontan state increase ET-1 transcription. Hypoxia induces stabilization of hypoxia-inducible factor-1α and upregulates EDN1 in endothelial and smooth-muscle cells (14, 15). Proinflammatory cytokines such as tumor necrosis factor-α and interleukin-1β, together with oxidative stress, activate NF-κB and AP-1 pathways to enhance ET-1 gene expression and peptide release (16–20). Shear forces are also important. Laminar pulsatile shear suppresses ET-1 and sustains nitric oxide activity, whereas the loss of pulsatility characteristic of the Fontan circulation removes this inhibitory influence (1, 11).

### Crosstalk with other vasoactive systems

ET-1 interacts with multiple vasoactive networks. Angiotensin II stimulates ET-1 production, and ET-1 in turn enhances angiotensin receptor signaling, creating a positive feedback loop (21, 22). Synergistic activity between serotonin and ET-1 has been demonstrated in promoting pulmonary arterial smooth-muscle proliferation (23). ET-1 can also augment sympathetic tone and induce angiogenic programs, linking vasoconstriction with vascular remodeling (24, 25). Although Fontan-specific studies remain limited, these mechanisms likely contribute to pulmonary vascular maladaptation in this setting (1–3).

### Processing and biomarker considerations

ET-1 is produced as prepro-ET-1, processed to big ET-1, and cleaved by endothelin-converting enzyme into the active peptide. The half-life of mature ET-1 in plasma is short, whereas big ET-1 and related fragments are more stable and reproducible for measurement (4, 26, 27). In chronic heart failure, elevated big ET-1 has been associated with adverse outcomes (26). In cohorts with single-ventricle physiology, higher ET-1 levels correlate with hospitalization and reduced functional capacity independent of systolic function (6). Within the Fontan circulation, impaired clearance due to reduced endothelial ET_B_ activity and hepatic congestion likely contribute to elevated circulating ET-1. As a result, big ET-1 represents a promising biomarker for trial enrichment, although its role requires validation in Fontan-specific cohorts before clinical application can be established (4, 7, 26, 27).

## Evidence for ET-1 activation across the fontan lifespan

### Perioperative kinetics and early adaptation

Immediately after Fontan completion, circulating ET-1 levels increase and correlate with pulmonary vascular resistance (28). Higher perioperative ET-1 has also been linked to elevated central venous pressure and prolonged pleural effusions, suggesting that the abrupt loss of pulsatility and increased afterload favor ET-1–mediated vasoconstriction (29, 30). The long-term implications of these acute elevations remain uncertain. There are limited data on ET-1 kinetics after the bidirectional cavopulmonary (Glenn) shunt. Glenn physiology is associated with increased central venous pressure and early hepatic congestion, and several cohorts demonstrate abnormal liver imaging and laboratory markers at this stage, but dedicated studies of circulating ET-1 are lacking (2, 31–34). Whether ET-1 activation begins during the Glenn phase and contributes to subsequent pulmonary vascular or hepatic remodeling remains an important unanswered question.

### Pediatric cohorts

Children and early survivors of Fontan palliation often exhibit ET-1 levels that exceed those of age-matched controls. In single-ventricle cohorts, elevated ET-1 has been associated with increased hospitalization and reduced 6-min walk distance independent of ventricular systolic function (6, 31). These findings imply that ET-1 reflects endothelial and pulmonary vascular stress rather than intrinsic ventricular dysfunction, although prospective pediatric data remain limited.

### Adolescent and adult trajectories

In adolescents and adults, ET-1 frequently remains elevated with increasing time from Fontan completion. Higher circulating levels are associated with worse functional class, diminished exercise tolerance, and greater arrhythmia burden (31–33). Preliminary data suggest that ET-1 trajectories may parallel noninvasive surrogates of pulmonary vascular resistance and hepatic stiffness, but longitudinal studies with rigorous phenotyping are needed.

### Endothelial activation signatures

Adults with Fontan physiology demonstrate circulating markers of endothelial activation beyond ET-1, including angiopoietin-2, which correlate with morbidity and reinforce an endothelial-injury paradigm (2, 33). These biomarkers may complement ET-1 in risk stratification and trial enrichment.

### Histopathologic evidence

Pulmonary tissue from failed Fontan circulations demonstrates overexpression of ET-1 and both ET_A_ and ET_B_ receptors in small pulmonary arteries, accompanied by medial hypertrophy and smooth-muscle proliferation (5). In pre-Fontan candidates, Aoki et al. reported that marked medial hypertrophy coexisted with strong ET-1 immunoreactivity, whereas patients without hypertrophy had lower ET-1 signal and more favorable outcomes; EDN1 upregulation by RT-PCR was observed in some with suboptimal postoperative courses despite reassuring resting hemodynamics (29). In explanted lungs from failed Fontan circulations, Ishida et al. confirmed ET-1 and receptor overexpression within intra-acinar arteries, closely resembling pulmonary arterial hypertension (5). Taken together, pathology-based data indicate that ET-1 is not merely a marker of endothelial stress but a participant in pulmonary vascular remodeling.

### Early interventional experience

Small, uncontrolled reports suggest that ERA therapy may improve borderline pulmonary vascular physiology in candidates for staged right-sided bypass. Hirono et al. described bosentan-associated hemodynamic improvement enabling successful Fontan completion in selected high-risk children (35). Similar anecdotal observations have been reported in other cohorts (36–38). Although limited to individual case reports, such as Wittczak et al., interventional experience mainly illustrates the creativity and technical feasibility emerging for end-stage Fontan failure. These observations remain anecdotal and should be interpreted with caution. These early experiences are summarized alongside clinical trial data in Table 1.

**Table 1:** Clinical trials of endothelin-pathway antagonists in the Fontan circulation.

Trial/study	Population (n)	Intervention & duration	Primary endpoint	Key results	Translational implications
TEMPO [Hebert et al. (36)]	39 adolescents & adults (mean age ∼19 year)	Bosentan, 14 weeks	Peak VO_2_	↑ Peak VO_2_ (+1.4 mL/kg/min), ↑ exercise duration, improved WHO class; no hepatotoxicity with monitoring	Proof-of-concept: ERAs can improve functional capacity in selected patients
Bosentan RCT [Schuuring et al. (37)]	26 adults	Bosentan, 6 months	Peak VO_2_	No significant change in VO_2_; secondary outcomes (exercise duration, QoL) trended favorable	Highlights heterogeneity of response; importance of phenotype selection
Ambrisentan crossover [Cedars et al. (38)]	15 adults	Ambrisentan, 12 weeks (crossover)	Peak VO_2_	↑ Peak VO_2_ and ventilatory efficiency; PK showed ↓ clearance vs. controls; generally tolerated	Demonstrates pharmacokinetic considerations in congested Fontan liver; supports safety
RUBATO Phase 3 [Clift et al. (39)]	211 adolescents & adults	Macitentan, 52 weeks	Peak VO_2_	Neutral primary endpoint; neutral most secondary outcomes; excellent hepatic safety, low discontinuation	Largest ERA trial to date; negative primary underscores need for enriched endpoints
RUBATO extension [Clift et al. (40)]	170 continued patients	Macitentan, 2 + years	Safety, exploratory endpoints	Sustained tolerability, no new hepatic signal; efficacy exploratory only	Confirms safety for chronic use; supports future precision-based ERA trials
Meta-analysis [Agnoletti et al. (41)]	4 trials, 118 patients	ERAs pooled	Variable (VO_2_, exercise)	Pooled ↑ in exercise parameters; heterogeneity noted	Early pooled evidence supports ERA benefit in selected phenotypes
Real-world registry [Constantine et al. (42)]	427 Fontan patients	ERA and/or PDE5i	Real-world outcomes	ERA use associated with improved submaximal exercise; safety consistent with trials	Confirms feasibility outside RCTs; underscores combination therapy
FUEL trial [Goldberg et al. (43)]	400 adolescents	Udenafil (PDE5i), 26 weeks	Peak VO_2_	Neutral VO_2_; improved VE/VCO_2_ slope, anaerobic threshold, O_2_ sat	Reinforces submaximal endpoints as physiologically aligned for Fontan

## Clinical trials of endothelin-pathway antagonism

### Bosentan

The TEMPO trial evaluated bosentan in adolescents and adults with Fontan physiology. Over 14 weeks of therapy, patients experienced improvements in peak oxygen consumption, exercise duration, and functional class without significant hepatotoxicity under protocolized monitoring (36). A smaller randomized trial in adults did not show significant improvement in peak oxygen consumption at six months, although secondary measures showed favorable trends (37). These differing results likely reflect heterogeneity of physiology.

### Ambrisentan

A double-blind crossover trial investigated ambrisentan in adults with Fontan circulation. Treatment was associated with improvements in peak oxygen consumption and ventilatory efficiency. Pharmacokinetic analyses revealed reduced clearance compared with non-Fontan populations, consistent with impaired hepatic extraction, but overall tolerability was acceptable (38).

### Macitentan

The 52-week phase 3 RUBATO trial assessed macitentan in Fontan patients. The study was neutral on its primary endpoint of peak oxygen consumption versus placebo, and most secondary outcomes were also neutral. Macitentan was generally well tolerated, with an excellent hepatic safety profile and low discontinuation rates (39). Exploratory analyses were hypothesis-generating and should be interpreted cautiously; efficacy was not demonstrated in prespecified subgroups. Long-term extension data confirmed continued tolerability (40).

### Related therapies and pooled signals

Pooled analyses combining ERAs with nitric-oxide–cGMP augmentation therapies report consistent improvements in submaximal exercise parameters such as ventilatory efficiency (VE/VCO_2_ slope), anaerobic threshold, and oxygen saturation, while effects on peak oxygen consumption remain variable (41, 42). The FUEL trial illustrates this: udenafil improved ventilatory efficiency and anaerobic threshold despite neutral results for peak oxygen consumption (43). An overview of the major clinical trials of endothelin receptor antagonists in Fontan patients is presented in Table 1.

## Why peak VO_2_ can be a misaligned endpoint

### Fontan-specific physiology

Peak oxygen consumption reflects maximal aerobic capacity in biventricular circulations, where stroke volume and heart rate both rise. In the Fontan circulation, absence of a subpulmonary ventricle constrains preload reserve, while elevated venous pressures further limit forward flow. Chronotropic impairment, reduced skeletal muscle mass, impaired mitochondrial function, and diminished oxygen extraction compound these limitations (44–48).

### Submaximal indices and clinical relevance

Submaximal measures provide a more physiologically aligned assessment of gas-exchange efficiency. The VE/VCO_2_ slope reflects ventilatory inefficiency and dead-space ventilation. Ventilatory or anaerobic threshold reflects improved pulmonary vascular reserve. The oxygen-uptake efficiency slope (OUES) is effort independent, while oxygen pulse and heart-rate recovery reflect stroke-volume augmentation and autonomic tone. These indices correlate with outcomes in congenital heart disease and respond to therapy when peak oxygen consumption does not (43, 45–48).

### Endpoint selection and implications

ET-1 constricts pulmonary resistance vessels and promotes vascular stiffening, limiting capillary recruitment during exertion. These changes increase central venous pressure and impede forward flow, compounding limited preload and impaired peripheral oxygen extraction. Impaired extraction has been identified as an independent prognostic factor in Fontan patients (44–47). Therapies targeting ET-1 or augmenting cGMP are therefore more likely to improve ventilatory efficiency and threshold measures than peak oxygen consumption, consistent with results from TEMPO, ambrisentan studies, and FUEL (36, 38, 43). Peak oxygen consumption remains a valuable contextual outcome but is best interpreted alongside submaximal indices including VE/VCO_2_ slope, ventilatory or anaerobic threshold, oxygen-uptake efficiency slope (OUES), oxygen pulse, and heart-rate recovery, and device-measured activity as primary or co-primary endpoints.(43, 45–48).

## Fontan-associated liver disease: where ET-1 biology intersects with congestion

### Venous hypertension and sinusoidal injury

Sustained elevation of central venous pressure combined with diminished hepatic pulsatility injures the sinusoidal microcirculation. Sinusoidal endothelial cells lose fenestrations, deposit basement membrane–like material, and upregulate adhesion molecules and vasoactive mediators (1, 33). Even modest increases in sinusoidal tone can accelerate congestion-related injury.

### Stellate-cell activation and fibrogenesis

Hepatic stellate cells are highly responsive to ET-1 through ET_A_ and ET_B_ receptors. ET-1 induces stellate-cell contraction, proliferation, and collagen synthesis via ERK and RhoA/Rho-kinase signaling (49–51). In experimental hepatic fibrosis, endothelin receptor blockade reduces fibrogenesis and collagen deposition. Human cirrhotic tissue studies localize ET-1 to activated stellate cells, where it increases contractility and collagen synthesis (49, 50). These mechanisms are biologically plausible in the congested Fontan liver, although direct confirmation is limited.

### Clinical implications and measurable endpoints

Management of FALD emphasizes hemodynamic optimization through arrhythmia control, valve repair, relief of obstructions, and fenestration strategies (2). Within this framework, ET-1–directed therapy may reduce sinusoidal resistance and slow fibrosis progression. Multiple elastography techniques, including transient elastography, point and two-dimensional shear-wave elastography, and magnetic resonance elastography, have been applied to Fontan cohorts (31–33). Across studies, these tools consistently detect higher liver stiffness in Fontan patients compared with non-Fontan controls and often show greater stiffness in patients with more advanced clinical or hemodynamic disease, as synthesized in systematic reviews of the available cohorts (31). In individual series summarized by Fathi et al., including pediatric data from DiPaola et al. and adult data from Bütikofer et al., liver stiffness increased after Fontan completion and was associated with markers of Fontan dysfunction, but there was substantial overlap between fibrosis stages and a clear influence of congestion on measured values (31). As emphasized in recent systematic reviews and expert statements, elastography reflects a composite of congestion and fibrosis and should not be interpreted as a stand-alone surrogate for histologic stage or global hepatic function (31–34).

Clinical studies that evaluate ET-1–directed approaches should prespecify hepatic endpoints that combine imaging and laboratory metrics. These could include liver stiffness measured by transient or shear-wave elastography and magnetic resonance elastography, platelet count as a surrogate for hypersplenism, spleen size, ascites burden, and biochemical markers such as *γ*-glutamyltransferase, bilirubin, and international normalized ratio (31–34). Work in adult Fontan cohorts, including multicenter studies and noninvasive biomarker analyses, supports the use of such multiparametric panels for risk stratification, although optimal thresholds and composite indices remain under investigation (31–34).

Data linking elastography to Fontan hemodynamics are emerging and should be interpreted with caution. Some studies report modest associations between liver stiffness and measures such as Fontan or central venous pressure and with composite clinical features of Fontan failure, but relationships are variable and confounded by dynamic changes in preload and congestion (31–33). Mori et al. demonstrated that central venous pressure was the hemodynamic variable most strongly associated with hepatic dysfunction, reinforcing a congestion–ET-1 positive-feedback loop (52). In this context, elastography is best viewed as a supportive tool for longitudinal surveillance and trial enrichment rather than a definitive measure of fibrosis. Serial changes in liver stiffness, interpreted alongside clinical status, laboratory indices, and cross-sectional imaging, may provide a practical way to assess the impact of endothelin-targeted therapies on hepatic congestion over time.

## Clinical pharmacology and safety

Fontan physiology alters hepatic drug extraction due to chronic congestion and portosystemic shunting. Bosentan, metabolized hepatically, carries a regulatory warning regarding hepatotoxicity (53), and this concern is echoed in developmental reviews of ERA pharmacology (54). In the TEMPO trial, however, no major hepatic safety signals were observed when liver function was monitored according to protocol (36, 53). Ambrisentan clearance is reduced in Fontan physiology, consistent with impaired hepatic extraction, but tolerability was generally acceptable, with mild anemia and fluid retention reported (38). Macitentan, with high tissue penetration and prolonged receptor occupancy, demonstrated favorable hepatic safety and low discontinuation rates in RUBATO, with only mild anemia and peripheral edema (39, 40).

Endothelin-receptor antagonists appear safe in Fontan cohorts when careful monitoring is applied. Future studies should integrate pharmacokinetic assessments and structured hepatic surveillance.

## Future directions

### Extending the pathway

Inhibition of endothelin-converting enzyme reduces ET-1 generation in preclinical pulmonary hypertension models and may complement receptor blockade, although Fontan-specific data are lacking (4). Pharmacologic strategies that selectively inhibit smooth-muscle ET_A_ and ET_B_ receptors while sparing endothelial ET_B_ could preserve vasodilatory and clearance functions. RNA-based approaches targeting EDN1 transcripts are conceptually attractive but remain experimental.

### Combination and adjunctive strategies

Because ET-1 activates hepatic stellate cells, combining ERAs with antifibrotic agents may better slow fibrosis than either approach alone. Mechanical or interventional strategies that reduce venous pressure, such as fenestration revision or surgical reconfiguration, should remain central, with medical therapy as adjunct.

### NO-independent cGMP stimulation

Impaired endothelial nitric oxide signaling and diminished pulsatility reduce endogenous cGMP activation in Fontan physiology. Soluble guanylate cyclase stimulators are effective in pulmonary arterial hypertension and chronic thromboembolic pulmonary hypertension (55, 56). Although untested in Fontan cohorts, they are biologically attractive in phenotypes with elevated PVR and ventilatory inefficiency.

### Exploratory genetics and pharmacogenomics

Genetic variability within the endothelin axis may contribute to the marked heterogeneity of outcomes and treatment response in Fontan patients. Polymorphisms in the EDN1 gene, particularly rs5370 (Lys198Asn), have been associated with hypertension and vascular risk in the general population, while other variants influence plasma ET-1 concentrations and vascular reactivity (26, 27). In systemic hypertension, these polymorphisms correlate with left ventricular hypertrophy and adverse remodeling, suggesting that ET-1–driven signaling cascades may be genetically modulated. Variations in endothelin receptor genes (EDNRA, EDNRB) and endothelin-converting enzyme (ECE1) have also been implicated in vascular tone regulation, pulmonary hypertension susceptibility, and endothelial dysfunction.

To date, no studies have systematically examined these variants in Fontan cohorts. Such data should be regarded as hypothesis-generating, but they provide a rationale for incorporating pharmacogenomic sampling into future Fontan trials. Beyond susceptibility, pharmacogenomic studies in pulmonary arterial hypertension suggest that genetic context may influence responsiveness to ERAs, a concept directly translatable to Fontan-directed therapies.

## A precision framework and pragmatic trial design

Therapeutic trials in the Fontan population should align interventions with phenotypes and employ endpoints reflecting underlying pathophysiology. Prospective enrichment should combine physiological markers such as elevated VE/VCO_2_, early ventilatory threshold, and low OUES with evidence of congestion or hepatic stiffness, as well as biomarkers of endothelial activation including ET-1, big ET-1, and angiopoietin-2 (4, 7, 26, 27, 31–33).

*Pulmonary-inefficiency phenotype:* Candidates include patients with abnormal VE/VCO_2_ slope, early ventilatory threshold, exertional desaturation, or catheter-confirmed pulmonary vascular load. These patients may benefit from ERAs alone or in combination with PDE5 inhibitors. Endpoints should include ventilatory efficiency, threshold measures, device-measured activity, and patient-reported outcomes (36, 38, 43, 45–48).
*Congestive-hepatic phenotype:* Characterized by evidence of hepatic congestion and dysfunction, including increasing liver stiffness on elastography or magnetic resonance elastography together with thrombocytopenia, splenomegaly, ascites, or abnormal liver function tests. Endpoints should capture both hepatic and systemic dimensions, including elastography, MELD-Xi, platelet count, and quality-of-life scales (31–34, 53, 54).
*Lymphatic-dominant phenotype:* Protein-losing enteropathy and plastic bronchitis remain major Fontan complications. Interventional lymphatic procedures, including embolization and thoracic-duct decompression, are first-line therapy (35, 57). ERA therapy may serve as an adjunct when elevated pulmonary vascular resistance contributes (52, 58).
*Arrhythmia-dominant phenotype:* Managed primarily with rhythm control and ablation strategies. Endothelin-targeted therapies may be considered in patients who demonstrate concurrent pulmonary or hepatic involvement (2).

Design features that can strengthen future trials include biomarker-guided enrichment using ET-1, big ET-1, or angiopoietin-2; co-primary endpoints combining pulmonary efficiency and hepatic stiffness; and adaptive randomization with interim analyses to refine enrichment. Embedded pharmacokinetic and pharmacogenomic substudies may help explain variability in drug response (27, 34, 38). Safety monitoring should include prespecified hepatic thresholds and careful interpretation of transaminase fluctuations within the baseline variability of Fontan-associated liver disease (53, 54).

## Testable predictions

In biomarker-enriched cohorts, endothelin-receptor antagonist therapy will produce greater improvements in ventilatory efficiency and threshold measures than in peak oxygen consumption (36, 38, 43, 45–48).
Combination therapy that integrates endothelin-receptor antagonism with nitric oxide–cGMP augmentation will provide greater benefit on ventilatory efficiency endpoints than monotherapy (41–43, 55).
In patients with congestive-hepatic phenotypes, endothelin-receptor antagonism may plausibly reduce sinusoidal resistance and hepatic congestion, which could be reflected by stabilization of liver stiffness measurements over time when interpreted alongside clinical and biochemical markers, rather than as evidence of fibrosis regression (32, 33, 53, 54).
Enrichment strategies guided by ET-1, big ET-1, or angiopoietin-2 will identify patient subgroups more likely to respond to therapy and thereby increase observed effect sizes (4, 7, 26, 27).
Genetic variation in EDN1 will stratify pharmacodynamic response to endothelin-targeted therapy and may explain heterogeneity in treatment outcomes (27, 34).

## Limitations

Endothelin-1 is unlikely to represent the sole determinant of outcomes in the Fontan circulation. Inflammatory pathways, lymphatic failure, arrhythmogenic substrate, and intrinsic myocardial disease also play important roles (2, 33). The evidence linking ET-1 to Fontan-associated liver disease is derived largely from studies of pulmonary hypertension and non-Fontan cirrhosis models, which limits direct applicability (49–51). Clinical trials of endothelin-receptor antagonists in Fontan cohorts remain modest in size, heterogeneous in patient selection, and variable in endpoint definitions and follow-up duration (36–40, 43). These constraints underscore the need for larger, prospective, phenotype-enriched studies with harmonized endpoints and mechanistic substudies to clarify causal pathways.

## Conclusions

Endothelin-1 provides a biologically coherent link between the hemodynamic stresses inherent to the Fontan circulation and cellular programs of vasoconstriction, vascular remodeling, and fibrogenesis. Clinical trials of endothelin-receptor antagonists have demonstrated reassuring safety. Signals of efficacy have been most evident when patients are selected for ET-1–driven inefficiency and when outcomes emphasize ventilatory efficiency or hepatic biomarkers rather than peak oxygen consumption alone (36–43, 53, 54). Future investigations should incorporate dual-organ trial designs, biomarker-guided enrichment, pharmacokinetic and pharmacogenomic substudies, and rational combinations with nitric oxide–cGMP augmentation or antifibrotic strategies.

Positioning ET-1 within Fontan pathobiology also reinforces the importance of multidisciplinary care. Effective integration of cardiology, hepatology, radiology, interventional, and genetic expertise will be required to translate ET-1–directed therapies from concept to clinic. Embedding biomarker testing, elastography, and genetic profiling within multidisciplinary Fontan programs may accelerate adoption of precision-based strategies and ensure clinically meaningful outcomes.

By reframing Fontan care through the lens of ET-1–driven biology, precision strategies emerge that may prolong stability, extend transplant candidacy, and improve long-term outcomes for this vulnerable population.

Summary of published trials of endothelin receptor antagonists (ERAs) and related therapies in Fontan patients. Trials include randomized controlled studies, crossover designs, and meta-analyses. Primary endpoints were generally peak oxygen consumption, while submaximal measures and safety outcomes provide complementary insights.
